# Do We Really Know How to Treat a Child with Bipolar Disorder or One with Severe Mood Dysregulation? Is There a Magic Bullet?

**DOI:** 10.1155/2012/967302

**Published:** 2011-12-01

**Authors:** Rajeev Jairam, Mukesh Prabhuswamy, Pravin Dullur

**Affiliations:** ^1^University of New South Wales, Kensington, NSW 2052, Australia; ^2^University of Western Sydney, Campbelltown, NSW 2560, Australia; ^3^Gna Ka Lun Adolescent Mental Health Unit, South West Sydney Local Health Network, Campbelltown Hospital, Therry Road, Campbelltown, NSW 2560, Australia; ^4^ICAMHS & Gna Ka Lun Adolescent Mental Health Unit, South West Sydney Local Health Network, Campbelltown Hospital, Campbelltown, NSW 2560, Australia

## Abstract

*Background*. Despite controversy, bipolar disorder (BD) is being increasingly diagnosed in under 18s. There is scant information regarding its treatment and uncertainty regarding the status of “severe mood dysregulation (SMD)” and how it overlaps with BD. This article collates available research on treatment of BD in under 18s and explores the status of SMD. *Methods*. Literature on treatment of BD in under 18s and on SMD were identified using major search engines; these were then collated and reviewed. *Results*. Some markers have been proposed to differentiate BD from disruptive behaviour disorders (DBD) in children. Pharmacotherapy restricted to short-term trials of mood-stabilizers and atypical-antipsychotics show mixed results. Data on maintenance treatment and non-pharmacological interventions are scant. It is unclear whether SMD is an independent disorder or an early manifestation of another disorder. *Conclusions*. Valproate, lithium, risperidone, olanzapine, aripiprazole and quetiapine remain first line treatments for acute episodes in the under 18s with BD. Their efficacy in maintenance treatment remains unclear. There is no validated treatment for SMD. It is likely that some children who are currently diagnosed with BD and DBD and possibly most children currently diagnosed with SMD will be subsumed under the proposed category in the DSM V of disruptive mood dysregulation disorder with dysphoria.

## 1. Introduction

A popular myth in psychiatry was that bipolar disorder is easily recognisable with clear episodes of mania and depression interspersed with lengthy periods of normal functioning. And that even when it onsets in children and adolescents it is straightforward to diagnose and can be treated with the same concoction of mood stabilizers and antipsychotics that “cures or controls” adults with bipolar disorder (BD). This myth has been decimated by the mountain of literature that has accumulated on paediatric bipolar disorder (PBD) over the last two decades. Much has been written about the difficulties and controversies surrounding its presentation and diagnosis, including whether DSM IV criteria can be reliably applied to diagnose children with bipolar disorder, whether irritability rather than euphoria is the predominant mood state and whether these outbursts of pathological mood states fulfil the required duration criteria. The reported high rates of comorbidity of attention-deficit hyperactivity disorder (ADHD) and disruptive behaviour disorders (DBDs) with PBD are another contentious area [1–4]. Evidence is beginning to emerge on the longitudinal course of PBD when it onsets in childhood or adolescence. Recent methodologically sound studies endorse childhood onset bipolar disorder as a relapsing disorder enduring into adulthood [5–7]: little, however, is known with regards to management of these children. Most treatment trials assume that all children with PBD present with an acute manic episode and should be treated as such. Consequently the majority of evidence is for time-limited pharmacotherapy in adolescents [8]. There are gaps in our knowledge in how to treat younger children with BD and in effective psychological and social forms of treatment. A thorough examination of this question is not complete without devoting some attention to children with “severe mood dysregulation” (SMD) who at face value seem to share a lot of characteristics with BD not otherwise specified (BD NOS), and to see what works for them. Different research groups have had different definitions for BD NOS, and consequently these have different conversion rates to BD [4]. Recent research also suggests a lower conversion rate of SMD to the classic bipolar phenotype when compared to the narrow phenotype bipolar disorder in the longitudinal course [9]. Perhaps the solution to the PBD puzzle lies in the amalgamation of psychosocial and pharmacological strategies that can effectively “control” this disorder so that its impact on normal development can be limited and positive health promoted. This paper will first briefly examine diagnostic issues and information on the course and outcome of PBD, followed by a synopsis on the current status of “severe mood dysregulation” as an entity. We will then examine the current evidence with regards to both pharmacotherapy and psychosocial management of both PBD and SMD.

## 2. Methods

A search was conducted using PubMed, Embase, and Psychlit from 1990 through 2011 using the search terms *pediatric bipolar disorder*, *mania*, *children*, *treatment*, *antipsychotics*, *mood stabilizers*, and *severe mood dysregulation*. The search was limited to clinical trials published in the English language. Weight-age was given to articles based on its type (review, original research, meta-analysis), its source of funding and kind of study (open trial, double blind trial, whether randomized control or not), and recency. The references of all recent review articles on pediatric bipolar disorder were reviewed manually to limit studies being missed. Some nonpublished studies which were presented in conferences were quoted in view of their importance. Older studies of lithium in PBD were included owing to limited recent data. The identified articles were reviewed by the three authors of the study and the results collated.

## 3. Diagnosis of Paediatric Bipolar Disorder

Breaking free of existential issues, the presence of BD in children and adolescents is now accepted [10–12]. Several aspects of it however remain controversial; for example, “How common is it?” Few prevalence studies of BD in adolescents exist and the most commonly quoted prevalence figure is 1% [10, 13]. There are no known prevalence studies of BD in prepubertal children. Diagnosis of bipolar disorder in adolescents is subject to little controversy. However the same issue in prepubertal children seems a different kettle of fish altogether. Those children and adolescents that demonstrate traditional DSM IV or ICD 10 criteria for BD are relatively straightforward to diagnose [14, 15]. These are the ones with BD type I and those with BD type II who present with clear hypomanic episodes. It is usually the other group of children who generate diagnostic controversy. They usually have very poor emotional regulation with significant outbursts, poor frustration tolerance, insecure attachments, and a learned pattern that temper tantrums and threats succeed in getting them what they want. Although there is a high comorbidity with disruptive behavior disorders (DBDs), that disorder alone does not explain all of their symptoms. These are a difficult group of children that clinicians often struggle with and it is tempting to dismiss this group as not being representative of bipolar disorder. After all, they lack the characteristics that have long been traditionally thought to correlate with BD including a week of mania (or four days of hypomania), clear-cut episodicity with the episodes lasting weeks or months, and good interepisode functioning. It is crucial to differentiate manic/mixed symptoms from disruptive behavior disorders, difficult temperament, developmentally appropriate excitement, and fantasy and those that are accentuated owing to the presence of a chaotic psychosocial environment [16, 17].

Some features whose presence would prompt a clinician to suspect BD not otherwise specified are as follows. (i) Onset: BD is not a developmental disorder, it is usually possible on thorough assessment involving the child and caregivers to establish premorbid functioning and an onset of current mood symptoms, (ii) Lack of clear precipitants: the occurrence of severe mood episodes appears to be internally driven rather than as a response to psychosocial precipitants, (iii) Unpredictable and unstable mood: which is a hallmark of a severe affective disorder like BD, (iv) Cycles of irritability, depression, elation and grandiosity lasting at least 4 hours—longer than could be explained by regular temper tantrums. In addition, these children have a chronic course with the mood disturbance sometimes lasting years, their mood cycles several times a day, and this type of BD has a younger average age of onset than the more traditional form. A positive family history of mood disorder is another common finding in this group of children [7, 12]. A landmark study which has clarified this further is the COBY study in which a four-year followup of 141 children with BD NOS revealed a 40% rate of conversion to BP I or II. They concluded that subsyndromal DSM-IV criteria may be valid to define BD NOS which is a strong predictor of BP I or II [7].

## 4. Outcome of Paediatric Bipolar Disorder

Available outcome studies of this group of children paint a bleak picture [5–7]. Studies on the course of BD which onsets in childhood and adolescence have yielded differing results. One set of studies show it to have a classical episodic course, with episodes lasting few months [18, 19] whereas others have found it to be more protracted with episodes lasting years [6, 20].

There have been five major retrospective studies over last two decades which compared adolescent onset with adult onset BD and found that those with adolescent onset had longer episodes, more number of episodes, greater comorbidity, longer time of illness before treatment, more rapid cycling, more suicidality, and poorer overall outcome [21–25]. Key prospective studies of children and adolescents with BD to date include two Indian and five American studies which have had sample sizes ranging from 25 to over 400 children with a one-to-eight year duration of followup [6, 7, 19, 26–28]. Overall these studies found that, with treatment, the mean index manic/mixed episode length was 10–14 weeks, 81–100% have episodic recovery, 50–80% experience one or more recurrences over a 5-year period, and the majority of recurrences occur in the first two years of the index episode. In addition over the duration of the study period, there was a switch from BP-NOS to BP I or II disorder and from BP II to BP I and the trend was child-adolescent-adult = BP NOS to BP I. In addition the studied young people had high rates of hospitalisation and health service utilization, high unemployment, legal problems, and poor academic and psychosocial functioning. Predictors of poor outcome included early onset, low socio-economic status, subsyndromal mood symptoms, longer duration of illness, BP NOS (rather than types I or II), other psychiatric comorbidities, and presence of significant family psychopathology. These findings however should not be overinterpreted as there have been few studies with small sample sizes with relatively short duration of followup as compared to adults with BD who have been followed up over a few decades, the studies used different methodologies and definitions of BD, and most studies recruited mainly BP I population. Further, the subjects entered various studies at different stages of illness, and there were different cultural and ethnic groups [5].

## 5. Pharmacological Treatment of Paediatric Bipolar Disorder

In stark contrast to adult research, there is limited data on the treatment of bipolar disorder in children and adolescents. Evidence is predominantly from open-label trials and few randomized control trials (RCTs). The American Academy of Child and Adolescent Psychiatrists came up with practice parameters in 2007 [29]. There is some evidence for the use of mood stabilisers including lithium and anticonvulsants, and antipsychotics (APs)—both typical and atypical in PBD and limited data available on other treatments. The only FDA-approved mood stabiliser for PBD is lithium (age 12 and older). FDA-approved antipsychotics are aripiprazole, risperidone, and quetiapine to treat acute manic and mixed episodes in BD aged 10–17 years. Olanzapine has been approved for BD aged 13–17 years. Aripiprazole was also approved for maintenance treatment in PBD aged 10–17 years. In Australia, no psychotropic medication is approved for use in persons under age of 18 years.

### 5.1. Synopsis of Key Studies on the Treatment of PBD

Kafantaris et al. used lithium and AP (haloperidol/risperidone) for treating acute bipolar mania in adolescents and noted lower relapses if the AP was maintained for at least 4 weeks [30]. Another study attempted lithium discontinuation after patients were acutely stabilised on lithium by randomising to lithium or placebo. As relapse rates in both the groups were high, a question of whether lithium is efficacious over a period of time was raised [31]. Yet another open-label study by this group demonstrated good efficacy in patients treated with lithium but noted that many patients were also on antipsychotics. The study did not clarify to what degree the response could be attributed to lithium [32]. Other trials have looked at the efficacy of lithium compared to valproate/placebo or lithium versus valproate/carbamazepine [33, 34]. Both these trials which were RCTs demonstrated better results with valproate than lithium or carbamazepine. However, a subsequent trial did not demonstrate any superiority for valproate over placebo [35]. Given the good efficacy in adults and the lack of strong methodologically rigorous studies on lithium, definitive studies pertaining to the efficacy in acute and long-term treatment of PBD (the Collaborative Lithium Trials (CoLT)) are currently in progress [36]. Furthermore, two trials of oxcarbamazapine did not support any significant benefit [37, 38]. Biederman et al. noted a significant improvement in the treatment of acute mania with Lamotrigine in a 12-week open-label trial of 39 adolescents [39]. However, this study had a higher dropout rate due to side effects especially skin rash.

Based on current evidence of mood stabilizer monotherapy for PBD, valproate has some evidence in controlled trials, while the evidence for lithium seems to come from open studies. Carbamazepine did not fare well.

Two studies evaluated lithium-valproate combinations in the acute treatment of bipolar mania or mixed states. One showed that patients with acute PBD depression or manic type respond to lithium-valproate combination therapy [40] and the other showed that most youth who had been stabilised on a combination therapy, who then relapsed on monotherapy, could be restabilised on combination therapy [41].

The most recent studies have tended to evaluate use of atypical antipsychotics in the treatment of acute bipolar mania. Pavuluri et al. compared risperidone to valproate in 66 patients and noted that risperidone was slightly faster acting and that group tended to have a better retention rate [42]. Another study found that quetiapine was as effective as valproate and faster acting in the acute manic phase of PBD [43]. Wozniak et al. noted that a combination of olanzapine and topiramate was no better than olanzapine monotherapy for acute mania in adolescents [44].

There have been few short-duration trials with other antipsychotics. Haas et al. noted that risperidone (0.5–2.5 mg/day) was efficacious and relatively well tolerated in the acute treatment of manic or mixed episodes in children and adolescents in the ages of 10 to 17 years [45]. In another RCT, 161 adolescents with an acute manic or mixed episode, Olanzapine (2.5–20 mg/d) was superior to placebo after 3 weeks [46]. Delbello et al. concluded that quetiapine, at doses of 400 and 600 mg/d, was more effective than placebo in treating acute manic symptoms in children and adolescents with BD in the ages of 10 to 17 years. Although the study duration was short (3 weeks), the sample size was large (277) [47]. Findling et al. (102) noted that Aripiprazol was effective in PBD (manic or mixed subtype) at doses of 10 mg/day and 30 mg/day [48]. Ziprasidone (dose 80–160 mg/day) was found to be superior compared to placebo in 238 adolescents aged 10 to 17 [49]. However, in April 2010, the FDA cited that the drugmaker had failed to properly ensure monitoring of its study, and as a result, widespread overdosing of patients at multiple study sites was neither detected nor corrected in a timely manner. So far, Ziprasidone has not been approved for treatment of PBD [50].

Very few studies evaluated maintenance therapy in PBD. Some studies have commented on the efficacy of lithium in preventing relapse [26, 51]. Findling et al. noted that both lithium and valproate were good maintenance agents but noted that, in both trials, symptoms returned subsequently in about 16 weeks [52]. Another study noted that aripiprazole was superior to placebo at 30 weeks [53]. Superiority of AP in maintenance treatment has not been demonstrated, and increased adverse events are observed with long-term treatment. Recommendations for MS (alone or in combination with AP) will be more accurate for maintenance therapy based on some studies; however there is still need for more research in this field.

Bipolar depression is probably the least studied of all the bipolar presentations in children and adolescents. Patel et al. found significant improvement for treatment of bipolar depression with lithium in an open-labelled trial [54]. Chang et al. noted that 63% were responders for lamotrigine in another open-label trial of paediatric depression [55]. Delbello et al. noted no difference in efficacy between quetiapine (300–600 mg/day) and placebo in a similar sample [56]. It is important to note that no difference was noted due to high rates of placebo response. There is a significant lack of studies on treatment of bipolar depression in children and adolescents, and more RCTs are needed to clarify the same.

In view of the vast data on the metabolic complications of atypical AP in paediatric patients, caution would be advocated in the use of APs in PBD [57]. One example was the study by Tohen which found significant weight gain and metabolic abnormalities in subjects on olanzapine [46]. Increased TG and cholesterol were noted especially with olanzapine and quetiapine and increased TG with risperidone and no significant metabolic changes with aripiprazole. It is important to note that the metabolic risk is heterogenous across AP. Maximum weight gain has been reported with olanzapine and least with aripiprazole [58, 59].

As no study has compared treatment efficacy between prepubertal and adolescent patients with PBD, recommendations for prepubertal patients cannot be made independent of adolescent patients. The limited data would suggest that, while acute mania in adolescents responds to either mood stabilisers or antipsychotics, short-term efficacy is probably better for antipsychotics than mood stabilisers [8]. Adult data suggest that the choice of mood stabiliser lithium versus valproate would depend on several factors. Some factors identified in adults with acute mania which favour a better response to lithium are euphoric mania rather than dysphoric, first episode mania, positive family history of bipolar disorder, positive family history of response to lithium, and absence of medical complications or substance abuse [60]. These factors need to be validated in PBD. Current data only supports the use of mood stabilisers for long-term maintenance, and the use of APs for this purpose should be pursued cautiously. The evidence for bipolar depression is even more limited but seems to reflect the adult guidelines which suggest first-line treatment should be lithium or lamotrigine [61].

## 6. Psychosocial Management of Paediatric Bipolar Disorder

Traditionally psychosocial interventions for PBD have focussed on psychoeducation of the illness, augmenting coping skills, and implementation of strategies to maximise medication compliance. As discussed earlier, the effectiveness of pharmacotherapy in returning a child with BD to premorbid functioning is unclear. In addition there are difficulties with compliance and with side effects of medications which may prevent a desired trial. As a result, there has been a renewed interest in evaluating the effectiveness of various psychosocial strategies in PBD, both as adjuncts and alternatives to pharmacotherapy and also in various stages of illness, right from those at high risk to develop PBD through to those who have had it for several years.

The feasible interventions that have been developed and have been received well by families in PBD include family-focused treatment (FFT), multifamily psychoeducation groups, interpersonal and social rhythm therapy (IPSRT), Cognitive Behavioural Therapy (CBT), and Dialectical Behavioural Therapy (DBT) [62].

Family-focussed therapy and multifamily psycho-education groups have demonstrated efficacy in alleviating mood symptoms, preventing recurrences, and enhancing psychosocial functioning in PBD. In addition, youth at high risk for BD including those with SMD have shown some benefit from targeted psycho-educational therapy or behaviour modification approaches and one study found that multifamily psychoeducation groups could exert a protective effect on conversion to BD among children with depressive spectrum disorders. [63–67]. The skill-training modules of FFT attempt to ameliorate the impact of high-EE attitudes and behaviours in families, including aversive communication between parent and offspring, hostility, low family cohesion and adaptability, and difficulties with conflict resolution [68]. A recent study examined one-year treatment outcome trial of an adapted version of FFT for youth (mean age 13.4 yrs) who were at high risk of developing BD-I or BD-II. These youth showed significant reductions in depression and hypomania symptoms and improvements in global functioning over one year [69]. There is consensus that psychosocial interventions need to focus on educating about signs of early relapse, adherence to treatment, coping strategies in interpersonal relations, and stress management. This can be achieved by using a combination of the above treatments. Stress management is a big part of psychosocial intervention since stress and trauma act as both contributing factors and as outcomes of manic/depressive episodes. There is evidence to suggest that young onset mania is triggered more often by stress than adult onset [70]. While there is more evidence in adults for the efficacy of these interventions, similar outcomes are starting to be reported in children and adolescents [71]. To date there is no data to suggest that any one form of psychosocial intervention is better than the other and all of them have been trialled in conjunction with pharmacotherapy. As with pharmacotherapy, the impact of psychosocial interventions on the social and academic functioning and quality of life of children with BD is unclear. Preventing or minimizing the toxic and impairing effects of BD early before they become chronic or recurrent may help prevent long-term functional disability.

## 7. Severe Mood Dysregulation (SMD)

### 7.1. Concept and Controversies

Rages, irritability, or anger outbursts which are out of proportion in both intensity and duration to the trigger can be a feature in several psychiatric and behavioural disorders in children and adolescents. SMD is a label that is often applied to prepubertal children with these rages. Other diagnoses that these children attract include major depression, oppositional defiant disorder (ODD), and attention deficit hyperactivity disorder (ADHD), Anxiety spectrum disorders and BD/PBD. SMD shares symptoms of severe irritability and hyperarousal seen in BD but the irritability is chronic and also the more common features of mania such as elation or grandiosity (as described in DSM-IV) are absent. Children with SMD exhibit developmentally inappropriate reactivity to negative emotional stimuli, such as “outbursts characterized by yelling and/or aggression,” which occur at least 3 times a week, and are impairing in at least 2 settings (home, school, peers). In addition, these children experience symptoms for at least a year without more than 2 symptom-free months. Onset is usually before age 12 with one study claiming an average age of onset of 5.1 years [72]. The DSM-5 task force has proposed the putative category of temper dysregulation disorder with dysphoria (TDDD) which has recently been changed to disruptive mood dysregulation disorder (DMDD) to describe children with severe irritability, and this is likely to replace SMD. DMDD is yet to be systematically studied but in essence is trying to describe the mood symptoms seen in children with SMD which is not described by just a diagnosis of ODD (and ADHD). Also it seems that DMDD diagnosis is not going to include the hyperarousal symptoms described in children with SMD; this would be a very heterogenous group with chronic irritability and recurrent severe temper outbursts. Owing to the ubiquitous nature of some of the proposed criteria, this diagnosis, if it makes it to DSM V, is likely to attract controversy [73]. The introduction of DMDD may however reduce the possible overdiagnosis of BD.

One of the biggest controversies in child mental health in the recent past has been the phenomenal increase in the prevalence figures for BD especially in the USA where a 500% increase has been reported [74]. Whether this is a fact or an artefact is a big debate in itself. What is relevant is whether rages or nonepisodic irritability is being diagnosed as “bipolar” (since irritability is considered to be more common than typical elation or sadness in early onset mood disorders) or if treatment options such as antipsychotics or mood stabilizers which are commonly prescribed for rages are driving a bipolar diagnosis. Other factors to consider are antecedent “benefits” of a “diagnosis” such as subsidised medication and access to supportive services.

Different research groups profess diverse views on the concept of SMD. Leibenluft et al. suggested these children with rages actually belong to a separate type of BD and compared the various clinical features, treatment, and family factors of these children with those with classical BD. The label SMD was borne out this effort which suggested that they are a different from the PBD group. Children with SMD also exhibit ADHD- and mania-like symptoms, including 3 of the following: insomnia, intrusiveness, and pressure of speech, flight of ideas/racing thoughts, distractibility, and psychomotor agitation. However, the severity of irritability and the intensity of mood/anxiety symptoms are greater in youths with SMD than those with ODD and ADHD. Biederman et al. suggest that the “SMD group” can be just subsumed under a combination of ADHD and ODD. However the ADHD plus ODD diagnosis fails to capture the mood and anxiety that characterize SMD youth. Carlson et al. suggested that it is important to recognize that a substantial proportion of children with this presentation have a learning disorder or a pervasive developmental disorder [75]. What contributions ODD, ADHD, and developmental disorders including autistic spectrum disorders make to the entity of SMD needs further study. The hope out of all of this is the eventual homogenization of this difficult-to-treat population and the development of treatment guidelines which can prevent the overprescription of mood stabilizers and other medications.

Recent studies have thrown more light on SMD. It is getting clearer now that these children are not as much at risk to develop BD as once thought or as one would intuitively presume. In fact, ADHD and depression have been more common in follow-up studies of this population [76, 77]. Family genetic studies have suggested that kids with SMD have family members with lower psychiatric morbidity rates than those seen in BD, ADHD, and ODD/CD [78]. This also points to SMD possibly being a separate entity and raises more questions about its genetic basis.

As evident from the above discussion there are several issues about SMD that are unknown and/or unclear. With its nosological status becoming clearer and with operationalized criteria in place one would hope to learn more about this group in the near future.

### 7.2. Treatment of SMD

What is known about treatment for SMD is mostly from expert views, clinical experience, and limited controlled trials. So far there are no specific validated treatments for SMD. The primary difficulty is in being able to define a relatively “homogenous” population. Almost all the drugs used in bipolar disorders have been trialled in SMD in addition to stimulants along with conjunct psychosocial therapies. Both lithium and divalproex sodium have shown some promise in this population [79] with one randomized placebo-controlled trial with lithium which showed a high placebo response (in the run-in phase) and also no significant difference between lithium and placebo in the randomized patients [80]. This needs to be studied in larger controlled studies. Treatment with antidepressants may need to be considered based on evidence from follow-up studies that major depression is a frequent outcome. We are some distance from being able to formulate treatment guidelines for SMD. With the introduction of operationalized criteria for DMDD and the advent of systematic studies, one would expect more robust literature on treatment of this challenging group of young people. However with the above discussion it appears that mood stabilizers do not seem to be the answer for children with SMD at this stage. It is important to tailor-make treatment for each child, use appropriate psychosocial interventions with active liaison with the school and all other stakeholders before using medications, treat ADHD appropriately, use medication like low doses of antipsychotics if arousal is the main problem, SSRIs if dysphoria is prominent (with careful monitoring for emerging suicidality or switch to mania), and have ongoing case management, the utility of which cannot be overstated. The potential risk of “creating” bipolar children with treatment especially antidepressants has to be kept in mind before initiating any pharmacotherapy. It is of utmost importance to make informed clinical decisions with the family once all these issues are discussed at length.

## 8. Summary

Controversy surrounding its morphology has not prevented PBD from being diagnosed in increasing numbers [81]. Attempts at defining a set of “criteria” to differentiate PBD from disorders such as DBD and SMD are being formulated. The precise manifestations of BD in prepubertal children will hopefully become clearer as more data on long-term outcome studies becomes apparent as it is this that will throw light on the early manifestations. The available long-term studies have been able to identify some predictors of outcome, and it does appear that the morphology of BD becomes more classical as the child grows older. Diagnostic controversy aside, treatment evidence for these children is extremely scant. Bulk of the studies have been pharmaceutical industry sponsored short-term trials of atypical antipsychotics and mood stabilizers. The available studies have significant limitations including small numbers, heterogeneity of sample (most studies of acute bipolar treatment include mania, hypomania or mixed) and short durations. Little is known about the benefits/efficacy of maintenance therapy or the acute treatment of bipolar depression. Lithium and atypical antipsychotics such as risperidone, olanzapine, aripiprazole and quetiapine remain first line treatments for acute episodes in PBD. Although their use is advocated in maintenance treatment, their efficacy remains unclear. Although commonly used, evidence supporting the use of sodium valproate in this population is lacking. Further trials with larger samples, longer duration, and homogeneity of diagnosis are clearly warranted. There is also a lack of well-researched psychosocial interventions for this population. Both family-focussed therapy and multifamily psycho-education appear to show promise. This area needs particular attention as medication alone is unlikely to be successful as a sole treatment in this developmentally crucial time. Other factors to consider are the presence of other comorbid psychiatric disorders, attachment difficulties, and family dysfunction which make diagnosis and treatment more complex.

SMD either as a symptom or as a disorder has generated recent interest. Although these children share some features of PBD, long-term outcome seems to suggest that these children may go on to develop depression with or without ADHD. The worry is that all those children who do not meet full PBD criteria are likely to receive the SMD diagnosis which is likely to make it another ragbag category. Effective treatment for SMD is likely to involve a combination of pharmacological and psychosocial treatment with significant involvement of the primary caregivers. However research to demonstrate treatment efficacy is still in its infancy. SMD is likely to be subsumed under the broad rubric of DMDD (DSM V) along with some of those currently diagnosed as PBD or DBD. There are already early signs that DMDD (previously TDDD) is not going to be without controversy either [73, 77].

PBD and SMD represent two diagnoses along the mood disorder spectrum that affects a subgroup of children. Tools to improve diagnoses, studies demonstrating long-term outcome, and longer treatment trials with both pharmacological and psychosocial therapies are the need of the hour.
